# The Effect of Roughness in Absorbing Materials on Solar Air Heater Performance

**DOI:** 10.3390/ma15093088

**Published:** 2022-04-24

**Authors:** Karmveer, Naveen Kumar Gupta, Md Irfanul Haque Siddiqui, Dan Dobrotă, Tabish Alam, Masood Ashraf Ali, Jamel Orfi

**Affiliations:** 1 Institute of Engineering and Technology, GLA University, Mathura 281406, India; karmsainiys@gmail.com (K.); naveen.gupta@gla.ac.in (N.K.G.); 2 Mechanical Engineering Department, College of Engineering, King Saud University, Riyadh 11421, Saudi Arabia; msiddiqui2.c@ksu.edu.sa (M.I.H.S.); orfij@ksu.edu.sa (J.O.); 3 Department of Industrial Engineering and Management, Faculty of Engineering, Lucian Blaga University of Sibiu, 550024 Sibiu, Romania; 4 CSIR-Central Building Research Institute, Roorkee 247667, India; tabishalam@cbri.res.in; 5 Department of Industrial Engineering, College of Engineering, Prince Sattam Bin Abdulaziz University, Al-Kharj 16273, Saudi Arabia; mas.ali@psau.edu.sa; 6 K.A.CARE Energy Research and Innovation Center, King Saud University, Riyadh 11421, Saudi Arabia

**Keywords:** roughness, solar air heater, thermal efficiency, Nusselt number and effective efficiency, augmentation

## Abstract

Artificial roughness on the absorber of the solar air heater (SAH) is considered to be the best passive technology for performance improvement. The roughened SAHs perform better in comparison to conventional SAHs under the same operational conditions, with some penalty of higher pumping power requirements. Thermo-hydraulic performance, based on effective efficiency, is much more appropriate to design roughened SAH, as it considers both the requirement of pumping power and useful heat gain. The shape, size, and arrangement of artificial roughness are the most important factors for the performance optimization of SAHs. The parameters of artificial roughness and operating parameters, such as the Reynolds number (Re), temperature rise parameter (ΔT/I) and insolation (I) show a combined effect on the performance of SAH. In this case study, various performance parameters of SAH have been evaluated to show the effect of distinct artificial roughness, investigated previously. Therefore, thermal efficiency, thermal efficiency improvement factor (TEIF) and the effective efficiency of various roughened absorbers of SAH have been predicted. As a result, thermal and effective efficiencies strongly depend on the roughness parameter, Re and ΔT/I. Staggered, broken arc hybrid-rib roughness shows a higher value of TEIF, thermal and effective efficiencies consistently among all other distinct roughness geometries for the ascending values of ΔT/I. This roughness shows the maximum value of effective efficiency equals 74.63% at a ΔT/I = 0.01 K·m^2^/W. The unique combination of parameters p/e = 10, e/D_h_ = 0.043 and α = 60° are observed for best performance at a ΔT/I higher than 0.00789 K·m^2^/W.

## 1. Introduction

Energy is the most important and serious matter worldwide. There are numerous energy sources out of which the most important and common source of energy is fossil fuel. The use of fossil fuels causes significant environmental problems due to which attention has been focused on renewable sources of energy. Solar energy is one of the best renewable energy sources because of its limitless energy, abundant quantity, cost-effectiveness, omnipresent reliability, easy-to-use, and non-pollutant nature all of the time. Solar energy has great potential to achieve the goal of sustainability due to these benefits.

**Figure 1:**
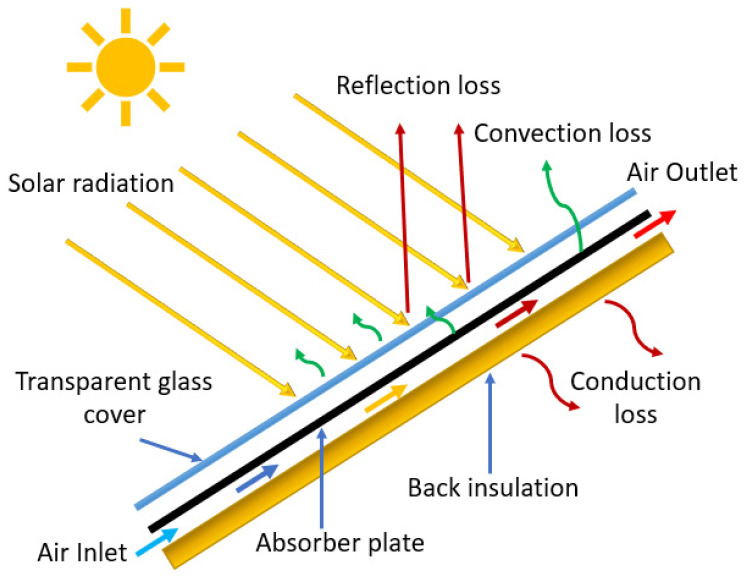
Basic illustration of a solar air heater (SAH).

SAH is known as the most attractive solar thermal system which can supply hot air for various purposes, ranging from domestic to industrial applications due to its simple design and cost-effectiveness [1,2]. However, SAH has poor performance due to the low convective heat transfer coefficient of the absorber plate and high heat losses from the top glass cover. Due to the development of the laminar sub-layer at the absorber plate, thermal resistance is generated near the plate which retards heat transfer. The convective coefficient can be augmented by using turbulators which induce turbulence in the duct near the absorber plate by disrupting and destabilizing the laminar sub-layer. The viscous sub-layer can be disrupted by using irregular shapes obstacles, called artificial roughness, in a distinct form of grits, grooves, baffles, ribs, winglets, protrusions, twisted taps, dimples, perforation and mesh wire, etc. On the other hand, the performance is also intrinsically affected due to the low heat capacity and thermal conductivity of flowing fluid [3]. To overcome these problems, distinct types of methodologies have been used by many researchers pertaining to achieve better improvement in the performance of SAH, such as using artificial roughness, extended surfaces, baffles, and porous surfaces. The basic illustration of a solar air heater (SAH) is presented in Figure 1.

The application of artificial roughness or turbulators, such as ribs, wires, sand grains, and metal grits on a heated duct surface, is an effective technique to augment the heat transfer rate to a flowing fluid [4]. 

In SAH, the aspect ratio of the duct is generally higher in comparison to other applications, while a range of Re and roughness to hydraulic diameter ratio, e/D_h_ are smaller in order to avoid the higher value of frictional losses and pumping power required to pump the flowing air [5].

The published literature on the application of roughness in SAH ducts covers a wide range of distinct types of roughness geometries, extended surfaces, fins, and protrusions [6]. Delta shape vortex generators were experimentally investigated with parameter P_l_/e = 3–5, P_t_/b = 0.6–1, e/D_h_ = 0.8, α = 45° and Re ranges from 2500–12,000. Results show augmentation in the Nusselt number, Nu, equal to 6.94 and also an increment in the friction factor equal to 45.83 over the smooth duct. The highest value of the thermal augmentation factor recorded was 2.26 [7]. Xio et al. [8] numerically studied the effect of inclined trapezoidal shape turbulators on the thermal and fluid flow performance characteristics of SAH. The longitudinal swirls are generated in the flow, which enhances the level of turbulation in the core region. The highest augmentations in exergy efficiency and energy efficiency are 31% and 24%, respectively, as a comparison to smooth duct. Dong et al. [9] numerically studied the effect of inclined groves’ ripple surface on the performance of SAH in the Re range of 12,000–24,000. This type of roughness also generated a combination of longitudinal swirl and secondary flow along with the fluid flow, which enhances turbulence. The secondary flow is generated in the inclined groves, which combine with the longitudinal swirls in the flow and further induce multi-counter rotating vortices in the vicinity of the flow. Nu is augmented by 1.54–6.96 and 1.21–3.38 times, as compared to a smooth surface case. The overall performance of the SAH also increases significantly. Jouybari and Lundstorm [10] studied the effect of porous material on the absorber surface. The highest increase in performance of SAH is about 5 times in comparison to a smooth surface with a minor increment in friction factor. The wall temperature of the duct reduces remarkably due to the implementation of porous material, resulting in a more uniform temperature across the heated surface. The value of THPP increased up to 5.5.

Momin et al. [11] experimentally studied the effect of V-shaped rib roughness on the performance of SAH. The roughness parameter, i.e., relative rib height (e/D_h_)_,_ the duct inclination angle (α) and Reynolds number vary in the ranges of 0.02–0.034, 30–90° and 2500–18,000, respectively, whereas p/e remains fixed at 10. The turbulence level near the heated surface increases due to reattachment, flow separation, and generation of a secondary flow. The V-shaped ribs are more advantageous than the inclined ribs. Momin et al. [11] reported that the value of Nu is 1.14 times more in comparison to inclined ribs, at Re equals 17,034. The highest augmentation in Nu and the friction factor are 2.30 and 2.83 times over the smooth duct at α = 60°. Istanto et al. [12] investigated the effect of continuous ribs of a V-down-shape type of roughness on the thermal characteristics of the SAH duct. The values of fixed-parameter are W/H = 12, p/e = 10, e/D_h_ = 0.033 and α varies from 30 to 80°. The Re ranges from 3500 to 10,000. They reported that the highest augmentation in friction factor and Nu were 2.45 and 2.34 times, respectively, over the smooth surface. The best value of THPP has been achieved at α equal to 60°. Singh et al. [13,14] investigated the effect of a V-down-shaped in discrete form of rib roughness on the performance of the SAH duct. The experimental parameters α, g/e, d/x, Re, e/D_h_ and p/e vary as 30–75°, 0.5–2.0, 0.20–0.80, 3000–15,000, 0.015–0.043 and 4–12, respectively. The maximum increase in the friction factor and Nu were reported as 3.11 and 3.04 times in comparison to the smooth duct at α = 60°. The effective efficiency of the SAH duct was enhanced up to 91% over the smooth duct. Deo et al. [15] analyzed the effect of a multi-gap V-down-shape rib combined with a staggered rib on the performance of the SAH duct. The following experimental parameter has been considered: e/D_h_ = 0.026–0.057, whereas ranges of the following parameters, α = 40–80°, Re = 4000–12,000 and p/e = 4–14 are considered. The value of fixed-parameter w/e = 4.5, g/e = 1 and p/P = 0.65. The highest augmentation in THPP and Nu were reported as 2.45 and 3.34 times, respectively, as a comparison to smooth duct. Hans et al. [16] studied the effect of multi-V-shape rib roughness on the performance of the SAH duct. The Reynolds number (Re), relative rib height (e/D_h_), width ratio (W/w), angle of attack (α) and relative rib pitch (p/e) varied in the range of 2000–2000, 0.019–0.043, 1–10, 30–75°, and 6–12, respectively. They also developed a correlation between Nu and friction factors by using an experimental parameter. They reported that the highest augmentation in Nu was found to be nearly 6 times over the smooth surface with some penalty due to an increase in the friction factor at α = 60°, e/D_h_ = 0.043, and p/e = 8. Singh et al. [17] investigated the effect of multi-V-shape rib with multi-uniform gaps on the performance of the SAH duct. They reported that the friction factor and Nu were augmented by 5.67 and 6.46 times, respectively, over the smooth duct at g/e = 1.0, e/w = 0.866, x/w = 0.25, W/w = 6, p/e = 10 and e/D_h_ = 0.0454. The highest value of THPP equals 4.24. They also developed correlations for Nu and the friction factor. Kumar et al. [18,19] studied the performance of artificial roughness having a V-shape with parameters W/e = 12, e/D_h_ = 0.0433, g/e = 1.0, W/w = 6, p/e = 10 and d/x = 0.69. The value of α varies from 30–75°. They reported that the friction factor and Nu are strong functions of α and have the highest value at α = 60°. They also developed correlations for Nu and the friction factor by using Re and various experimental parameters.

Yadav et al. [20] studied the effect of circular protrusion in angular arc patterns on the performance of SAH. The experimental parameter was considered in the following ranges of e/D_h_ = 0.015–0.03, p/e = 12–24, α= 45 to 75°, and Re= 3600–18,100. The highest increment in *f* and Nu was reported as 2.93 and 2.89 times, respectively, at α’= 60°, p/e = 12 and e/D_h_ = 0.03. They developed correlations for Nu and the friction factor by using experimental results and various experimental parameters. Hans et al. [21] experimentally studied the effect of broken-arc rib roughness on the performance of the SAH duct. The broken arc of the roughness contains uniform gaps. The experimental parameter varies as g/e = 0.5 to 2.5, d/w = 0.5 to 0.8, p/e = 4 to 12, α’ = 15–75°, e/D_h_ = 0.022–0.043, Re = 2000 to 16,000. They reported that the Nu was enhanced by 2.63 times as compared to the smooth duct at e/D_h_ = 0.043 and p/e = 10. Gill et al. [22] studied the effect of staggered broken-arc hybrid-rib roughness on the thermo-hydraulic performance of SAH. The ranges of the following parameters are given as e/D_h_ = 0.022–0.043, α = 15–75°, p/e = 4–12, g/e = 0.5–2.5 and Re = 2000–16,000. The *f* and Nu were augmented by 2.57 and 3.16 times over the smooth duct at e/D_h_ = 0.043. The Nu was enhanced by 1.22 times over the broken arc rib. The highest value of THPP has been reported as 2.33. 

Lanjewar et al. [23] experimentally studied the effect of W-shaped rib roughness both in W-down and W-up patterns on the performance of the SAH duct. The following roughness parameters, e/D_h_ = 0.03375, Re = 2300–14,000 and p/e = 10 are considered in these studies. The enhancing range of THPP equals 1.21 to 1.73 and 1.46 to 1.95, respectively, for top-up and top-down patterns. The W-down pattern has a better performance in comparison to the W-up pattern. The highest value of the thermo-hydraulic ratio for W-up and W-down patterns is 1.73 and 1.95, respectively. Kumar et al. [24] studied the effect of discrete-W-shaped rib on the thermo-hydraulic performance of SAH in the Re range of 3000–15,000. The value of parameter p/e is kept constant at 10, whereas α varies from 30° to 75°, e/D_h_ varies from 0.0168 to 0.0338 during the experiment. Nu increases with the increase in Re and the friction factor decreases. The highest augmentation in the friction factor and Nu were found to be 2.75 and 2.16 at e/D_h_ = 0.0338 and α = 60°. Gawande et al. [25] studied the effect of the reverse L-shaped repeated rib type of roughness on the convective performance of SAH through experimentation and CFD analysis. The following roughness parameters, i.e., p/e varies from 7.14 to 17.86 and Re from 3800 to 18,000. However, the value of e/D_h_ was kept constant at 0.042. The highest enhancement in Nu was found to be up to 2.827 times that of the smooth duct at p/e equal to 7.14, Re = 15,000, and e/D_h_ = 0.042. The THPP was found to be in the range of 1.92 to 1.90.

Kumar and Layek [26,27] studied the performance of SAH duct with winglet types of turbulators with a small hole at the tip. The experimental parameter covered the range of parameters as α equal to 30–75°, p/e equal to 5–12, W/w = 3–7 and Re = 3000–22,000. The optimum value of Nu was achieved at α = 60°, and p/e = 8. The Nu increases while the friction factor decreases with the increase in Re. Patel et al. [28] studied the effect of a reverse NACA 0040 type of profile rib roughness on the performance of SAH in the Re range of 6000 to 18,000. The following parameter kept constant p/e = 8 and e/D_h_ = 0.065. The THPP of 2.53 was achieved at Re = 6000. The maximum value of Nu of 104.45 was achieved at Re = 18,000. Kumar and Layek [29] also have conducted numerical analyses for optimizing the exergetic efficiency and effective efficiency by using the same type of rib roughness. The highest augmentation in effective efficiency, thermal efficiency, and exergetic efficiency are equal to 1.79, 1.81, and 1.81 times over the smooth duct at a twist ratio equal to 3 and p/e = 8. Promvonge et al. [30] experimentally studied the effect of V-shape rib and delta groove on the absorber plate, respectively. The Re varied in the ranges of 7000–30,000, whereas attack (α) was considered as 60° The highest value of thermal performance was achieved at e/H = 0.108 and p/H = 1.0.

The above-presented works have proposed and evaluated various types of solar air heaters encompassing numerous geometric extensions and obstructions, such as obstacles and ribs with different roughness, form, and material types. The central objective of these works is to enhance the heat transfer by, for example, surface extension and turbulence promotion. The obtained results are quite encouraging for some attempts. For others, the penalty of such modifications on the basic SAH seems to be high. The friction factor and the related pumping power are important. These works are generally undertaken using experimental approaches and/or lumped models by considering various types of correlations. CFD studies are also presented. The scientific relevance of these works to the heat transfer technical and scientific communities should be highlighted and the most important results and conclusions should be screened and retained for use and implementation. Therefore, a systematic comparative study of these works and related correlations and results is needed. This case study aims to establish a general framework of the most relevant and updated studies on the performance of SAH by exploiting various ribs configurations. The performance comparison of the various ribs in SAH has been presented and discussed, which would be helpful to researchers who are working in this area. 

## 2. Thermal and Effective Performance Assessment Approach of Roughened SAH

The useful heat gain (Q_u_) to the working fluid in terms of the mean temperature of the absorber plate can be evaluated as [31]:

(1) $$Q_{u}=\left[I{\left(\tau \alpha \right)}_{avg}-U_{o}\left(T_{pm}-T_{a}\right)\right]A_{p}$$

where (τα)_avg_ is the transmittance and absorbance product, U_o_ is the overall heat loss coefficient, T_pm_ is the mean temperature of the absorbing plate, and T_a_ is the ambient temperature. A_p_ is the aperture area. 

**Figure 2:**
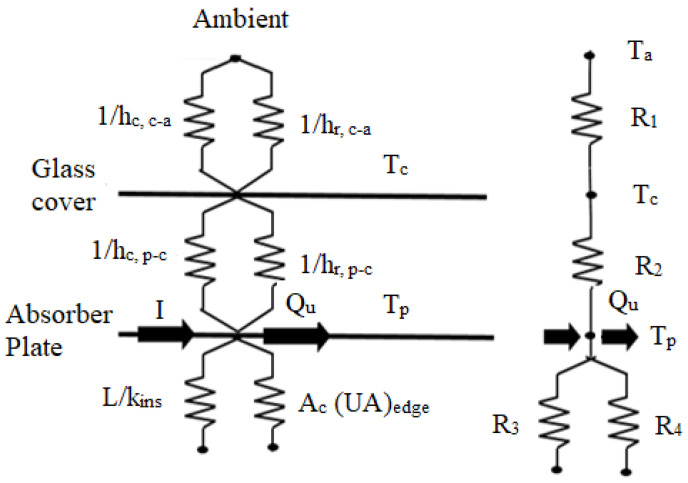
Thermal resistance in SAH on the basis of convection and radiation coefficient.

The overall heat loss coefficient is given by:

(2) $$U_{o}=U_{t}+U_{b}+U_{e}$$

where U_b_ is the back loss coefficient, U_t_ is the top loss coefficient, and U_e_ is the edge loss coefficient. Thermal resistance in SAH on the basis of convection and radiation coefficient is shown in Figure 2.

The heat transfer between the glass cover and absorber plate is:

(3) $$\begin{array}{l} q_{loss,p-c}=h_{c,c-p}\left(T_{p}-T_{c1}\right)+\frac{\sigma \left(T_{p}^{4}-T_{c}^{4}\right)}{\frac{1}{\varepsilon_{p}}+\frac{1}{\varepsilon_{c}}-1}=\left(h_{c,p-c}+h_{r,p-c}\right)\left(T_{p}-T_{c}\right) \end{array}$$

(4) $$h_{r,p-c}=\frac{\sigma \left(T_{p}^{2}+T_{c}^{2}\right)\left(T_{p}^{}+T\right)}{\frac{1}{\varepsilon_{p}}+\frac{1}{\varepsilon_{c}}-1}$$

(5) $$\begin{array}{l} R_{2}=\frac{1}{\left(h_{c,c-p}+h_{r,c-p}\right)} \end{array}$$

Heat transfer due to convection between the glass cover and the absorber plate:

(6) $$h_{c,p-c}=\frac{Nu\cdot k}{L}$$

Heat transfer due to radiation between the glass cover and the sky:

(7) $$h_{r,c-a}=\frac{\sigma \varepsilon_{c}\left(T_{c}^{}+T_{s}^{}\right)\left(T_{c}^{2}+T_{s}^{2}\right)\left(T_{c}^{}-T_{s}^{}\right)}{\left(T_{c}-T_{a}\right)}$$

The resistance to ambient:

(8) $$R_{1}{}_{}=\frac{1}{\left(h_{r,c-a}+h_{w}\right)}$$

The top loss coefficient from the glass cover to the surrounding is:

(9) $$U_{t}{}_{}=\frac{1}{\left(R_{1}+R_{2}\right)}$$

The edge loss coefficient is:

(10) $$U_{e}=\frac{1}{R_{4}}=\frac{{\left(UA\right)}_{edge}}{A_{p}}$$

The back loss coefficient is:

(11) $$U_{b}=\frac{1}{R_{3}}=\frac{k_{ins}}{T_{i}}$$

The thermal efficiency of the collector is:

(12) $$\eta_{th}=\frac{Q_{u}}{A_{p}I}$$

Cortes and Piacentini [32] defined the net effective efficiency of the solar collector on the basis of net heat gain and is expressed as:

(13) $$\eta_{net}=\frac{Q_{u}-\frac{P_{m}}{C}}{A_{p}\cdot I}$$

where *P_m_* is the mechanical power required by the blower to propel the air through the duct. It can be expressed as [33]:

(14) $$P_{m}=\frac{m\cdot \Delta P_{d}}{\rho_{a}}$$

where ΔP_d_ is the pressure drop across the duct. It can be expressed as [34]:

(15) $$\Delta P_{d}=\frac{4f\cdot L\cdot \rho_{a}\cdot V^{2}}{2d_{h}}$$

$C\left(=\eta_{p}\times \eta_{m}\times \eta_{Tr}\times \eta_{T}\right)$ is the conversion factor used for converting Pm into thermal energy. Cortes and Piacentini [32] recommended the value of C, which is 0.18, which is calculated based on the following efficiencies, (pump efficiency (η_p_) = 0.65, motor efficiency (η_m_) = 0.88, transmission efficiency (η_Tr_) = 0.925, thermal power plant efficiency (η_T_) = 0.344).

## 3. Mathematical Model for Calculation of Thermo-Hydraulic Performance of SAH

The heat transfer behaviour of the roughened SAH is similar to that of conventional SAH, as in both types of SAH insulations from the sun are absorbed by the collector plate and further heat is transferred to the working fluid. The absorption of insolation and various heat losses from the roughened SAH can be evaluated by using the same methodology as for conventional SAH. The thermo-hydraulic performance of SAH can be expected and analysed on the basis of a detailed consideration of thermal and hydraulic processes. The calculation of thermal and effective efficiency proceeds by using the value of the system and operating parameter as for conventional SAH. The range of system parameters has been decided on the basis of various experimental studies, and operating parameters have been decided on the basis of the application of SAH. The optimum thermal and effective efficiency has been calculated by using solar insolation’s (I) and the temperature rise parameter (ΔT/I). The distinct properties of air, i.e., thermal conductivity (k), dynamic viscosity (μ), specific heat (C_p_), and density (ρ) were calculated with the help of relations given by Bhushan and Singh [34] and Hans [35] as:

(16) $$k=0.0275\times {\left(\frac{T_{f}}{293}\right)}^{0.086}$$

(17) $$C_{p}=1006\times {\left(\frac{T_{f}}{293}\right)}^{0.0155}$$

(18) $$\mu =1.81\times 10^{-5}\times {\left(\frac{T_{f}}{293}\right)}^{0.735}$$

(19) $$\rho =\left(\frac{P_{a}}{RT_{f}}\right)$$

The procedure adopted for the calculation of thermal and effective efficiency is the same as it was given by Alam T et al. [36], and a MATLAB code has been generated for the same. The step-by-step calculation procedure of thermal and effective efficiency, as suggested by Bisht et al. [5], Sahu and Prasad [37], Yadav and Kaushal [38], Chamoli and Thakur [39], and Alam T et al. [36], is as follows: 

Step 1: Initialize values of the fixed system and operating parameter from Table 1. The p/e and e/D_h_ are the dimensionless geometrical parameters of rib for which effective efficiency is to be evaluated.

Step 2: The outlet temperature (T_o_) of the air is calculated with the help of solar insolation, inlet air temperature and temperature rise of air as:

(20) $$T_{o}=\left(\frac{\Delta T}{I}\right)\times I+T_{i}$$

The ambient air of the surrounding atmosphere is sucked by the SAH duct so, the inlet air temperature is considered to be equal to the surrounding temperature.

Step 3: The mean fluid temperature is calculated as:

(21) $$T_{mf}=\frac{T_{o}+T_{i}}{2}$$

Step 4: The value of mean plate temperature is presumed as:

(22) $$T_{mp}=\frac{\left(T_{o}+T_{i}\right)}{2}+10$$

Step 5: The thermo-physical properties of air are calculated with the help of correlations 14, 15, 16 and 17, given by Bhushan and Singh [34] and Hans [35].

Step 6: The overall heat loss coefficient (U_o_) is calculated as:

$$U_{o}{}_{}=U_{t}+U_{b}+U_{e}$$

where the top heat loss coefficient is calculated as [40]:

(23) $$\frac{1}{U_{t}}={\left[\frac{\sigma \left(T_{pm}^{2}+T_{g}^{2}\right)\left(T_{pm}+T_{g}\right)}{\left(\frac{1}{\varepsilon_{p}}+\frac{1}{\varepsilon_{g}}-1\right)}+\left(\frac{k_{a}Nu_{1}}{L_{g}}\right)\right]}^{-1}+{\left[\sigma \varepsilon_{g}\left(T_{g}^{2}+T_{a}^{2}\right)\left(T_{g}+T_{a}\right)+h_{w}\right]}^{-1}+\frac{t_{g}}{k_{g}}$$

where,

$$T_{g}=\left(\frac{F_{1}T_{pm}+cT_{a}}{1+F_{1}}\right)$$

where,

$$F_{1}=\frac{{\left[12\times 10^{-8}{\left(T_{a}+0.2T_{p}\right)}^{3}+h_{w}\right]}^{-1}+0.3t_{g}}{{\left[6\times 10^{-8}\left(\varepsilon_{p}+0.028\right){\left(T_{pm}+0.5T_{a}\right)}^{3}+0.6L_{g}^{-0.2}{\left\{\left(T_{pm}-T_{a}\right)cos\beta_{ti}\right\}}^{0.25}\right]}^{-1}}\;c=\left[\frac{\left(T_{s}/T_{a}\right)+\left(h_{w}/3.5\right)}{1+h_{w+3.5}}\right]\;T_{s}=0.0522{\left(T_{a}\right)}^{1.5}\;\begin{array}{c} Nu_{1}=1+1.44{\left[1-1708/RaCos\beta_{ti}\right]}^{+}\left\{1-1708{\left(sin1.8\beta \right)}^{1.6}/Racos\beta_{ti}\right\}\\ \;\;\;\;\;\;\;\;+\left[{\left(RaCos\beta_{ti}/5830\right)}^{0.33}-1\right] \end{array}\;Ra=Gr\times Pr\;G_{r}=\frac{g\beta^{\prime }\left(T_{pm}-T_{g}\right)L_{g}^{3}}{v^{2}}$$

The back cover heat loss coefficient is calculated as:

(24) $$U_{b}=\frac{k_{ins}}{T_{i}}$$

The edge heat loss coefficient is calculated as:

(25) $$U_{e}=\frac{\left(L+W\right)t_{e}k_{i}}{LWt_{i}}$$

Step 7: The useful heat gain is calculated as:

(26) $$Q_{u1}=\left[I\left(\tau \alpha \right)-U_{o}\left(T_{pm}-T_{a}\right)\right]A_{P}$$

Step 8: The mass flow rate is calculated as:

(27) $$m=\frac{Q_{u1}}{C_{p}\Delta T}$$

Step 9: The Reynolds number is calculated as:

(28) $$Re=\frac{mD_{h}}{\mu WH}$$

Step 10: The Nusselt number of different roughness surfaces has been estimated with the help of their developed correlations. Then, the convection coefficient of heat transfer has been estimated as:

(29) $$h=\frac{Nu\cdot k}{D_{h}}$$

The Nu for smooth surface has been estimated with the help of the Dittus–Boelter correlation as:

(30) Nu=0.023Pr0.4Re0.8

Step 11: Again, heat gain by the flowing air is evaluated by estimating the heat removal factor, collector fin efficiency as follows [32]:

(31) $$Q_{u2}=A_{p}F_{o}\left[I\left(\tau \alpha \right)-U_{l}\left(T_{o}-T_{a}\right)\right]\;F_{o}=\frac{mC_{P}}{A_{P}U_{l}}\left[exp\left\{\frac{F^{\prime }A_{P}U_{l}}{mC_{P}}\right\}-1\right]$$

$$F^{\prime }{}_{}=\frac{h}{h+U_{o}}$$

Step 12: Then, Qu_1_ and Qu_2_ are compared if, these values are not matched with each other, the new value of T_pm_ is determined by using the value of heat gain, Q_u2,_ from Equation (27). Iteration continues until the values of Q_u1_ and Q_u2_, come close enough.

(32) $$Q_{u2}=A_{p}F_{o}\left[I\left(\tau \alpha \right)-U_{l}\left(T_{o}-T_{a}\right)\right]\;T_{pm}=T_{a}+\left[\frac{I\left(\tau \alpha \right)-Q_{u2}/A_{p}}{U_{l}}\right]$$

Step 13: The friction factor of different roughness surfaces has been estimated with the help of their developed correlations. The friction factor of a smooth surface has been estimated with the help of the Blasius correlation as:

(33) f=0.079Re−0.25

Step 14: The mechanical power required by the blower is calculated with the help of Equation (14).

Step 15: The pressure drop across the duct has been calculated with the help of Equation (15).

Step 16: Final thermal efficiency and the effective efficiency of different roughness have been estimated with the help of Equations (12) and (13).

**Figure 3:**
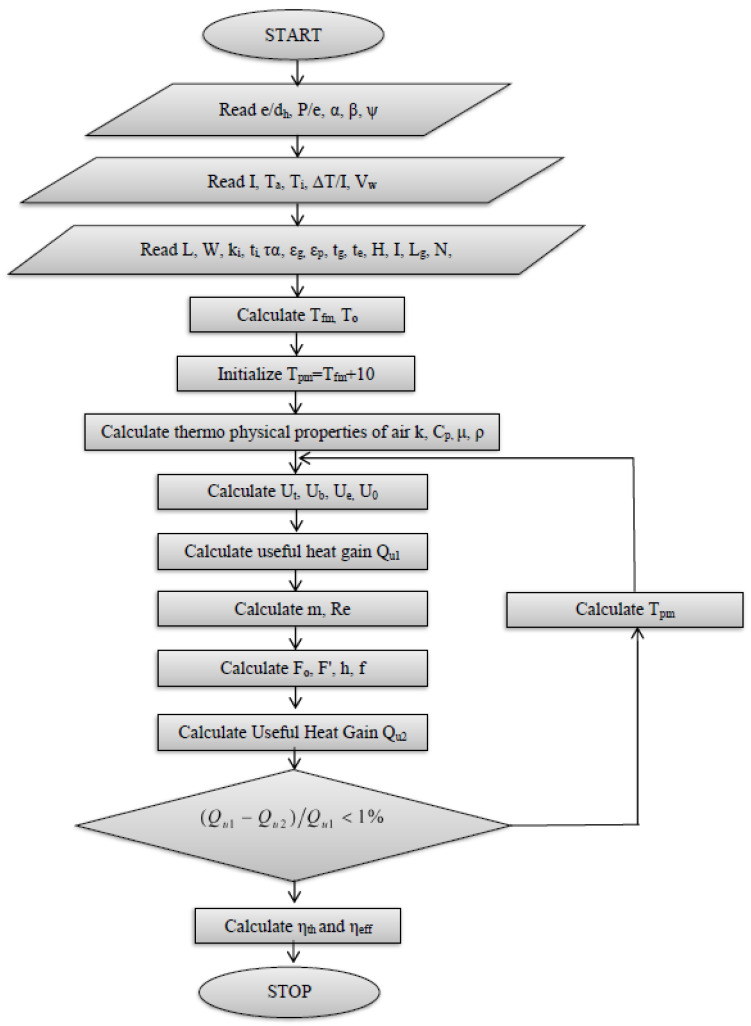
Flow chart of methodology.

The research methodology used is presented in Figure 3.

The correlations developed by researchers for different rough surfaces are presented in Table 2.

## 4. Discussion

The numerical analysis of the roughened duct of SAH with distinct types of roughness geometry and arrangements is carried out with the help of the methodology discussed in the previous section. The system and operating parameters with distinct types of roughness geometry and their arrangement are considered for estimating the thermo-hydraulic performance of SAH. The system and operating parameters are tabulated in Table 1 and correlations developed by the different researchers for different rib roughened surfaces are tabulated in Table 2. The plot of effective efficiency showed an indirect comparison of useful heat gains and pumping power requirements for various parameters as a function of Re and ΔT/I. The performance analysis of distinct types of roughness relies on thermal and effective efficiency. Then, the roughness having optimal performance is selected and compared with the others under the same parameters. The cover temperatures and the heat flux by convection and radiation are shown in the thermal network of SAH (Figure 2). The efficiency and overall heat loss coefficient of the SAH duct have been calculated.

It is very clear that the mean absorber plate temperature decreases with the increase in Re, in contrary to this, the heat gain increases with the increase in Re and becomes almost flat for a higher value of Re. This implies that the lower mean absorber plate temperature exhibits a high rate of heat extraction from the absorber plate by the air because of low heat losses from the glass cover. The pumping power requirement also increases slightly as Re increases, but the rate of increment is not too significant.

**Figure 4:**
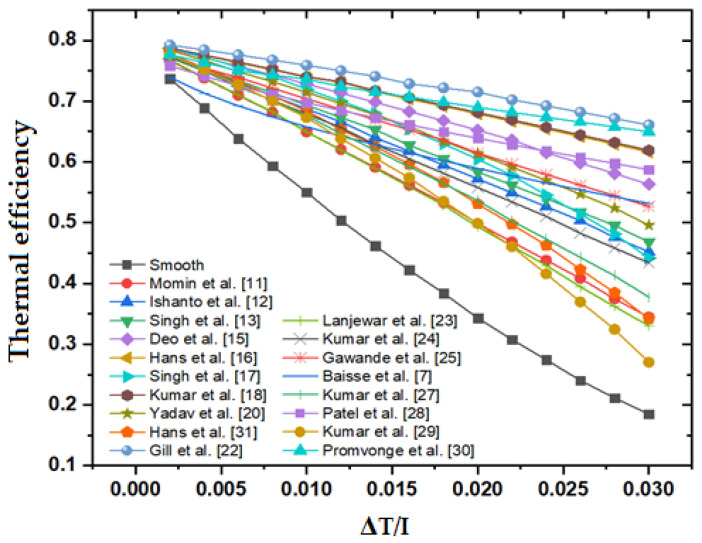
Thermal efficiency of the roughened duct with respect to ΔT/I.

**Figure 5:**
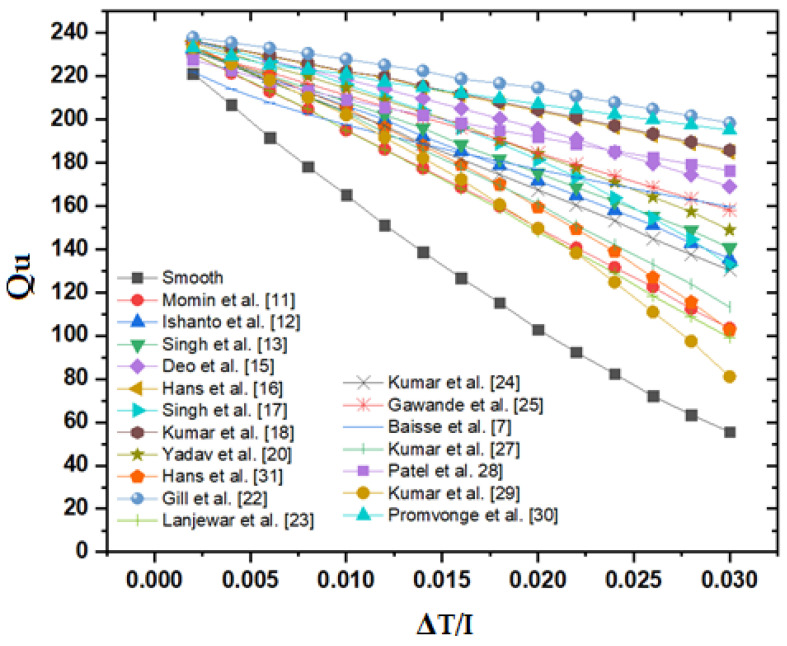
Useful heat gain of the roughened duct with respect to ΔT/I.

Figure 4 shows how the roughened duct’s thermal efficiency varies with ΔT/I and Figure 5 shows the useful heat gain of the roughened duct with respect to ΔT/I. Table 3 also contains information on thermal efficiency. The thermal efficiency of SAH for all various roughness geometries drops as the temperature rise parameter rises, although TEIF and the total heat loss coefficient increase. Bisht et al. [5] earlier documented a similar sort of trend in their graphic. This occurs when the temperature of the absorber plate rises, causing the heat to escape into the surroundings. The higher emissivity of the absorber plate at higher temperatures, on the other hand, is partly to blame for the increased heat loss to the environment.

The thermal efficiency of all roughness diminishes with increasing ΔT/I, as shown in the figure, and this pattern is comparable to that previously found by Chamoli and Thakur [39], and Alam T et al. [36]. At lower levels of ΔT/I, the influence of ΔT/I on thermal efficiency is insignificant, but at higher values of ΔT/I, the thermal efficiency plot of various roughness geometries diverges substantially, and a noticeable differentiation is noted among diverse roughness geometries. For increasing levels of ΔT/I, the staggered broken arc hybrid-rib roughness displays consistently superior values of thermal efficiency across different roughness geometries.

**Figure 6:**
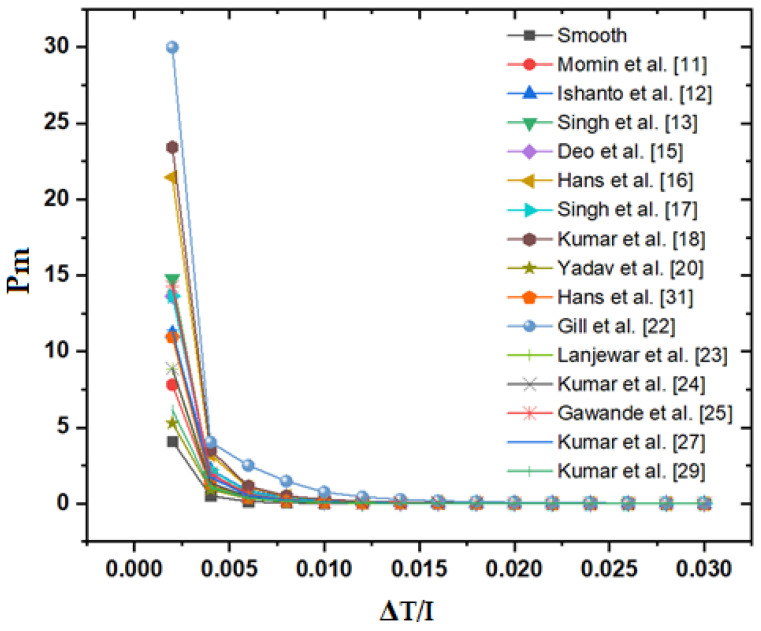
Pumping power requirement of the roughened duct with respect to ΔT/I.

The staggered broken arc hybrid-rib roughness shows consistently higher values of useful heat gain among distinct roughness geometries for the ascending values of ΔT/I. The plot between pumping power requirement and ΔT/I in all cases is shown in Figure 6. The staggered broken arc hybrid-rib shows a slightly higher value of pumping power requirement among distinct roughness geometries for the ascending values of ΔT/I.

**Figure 7:**
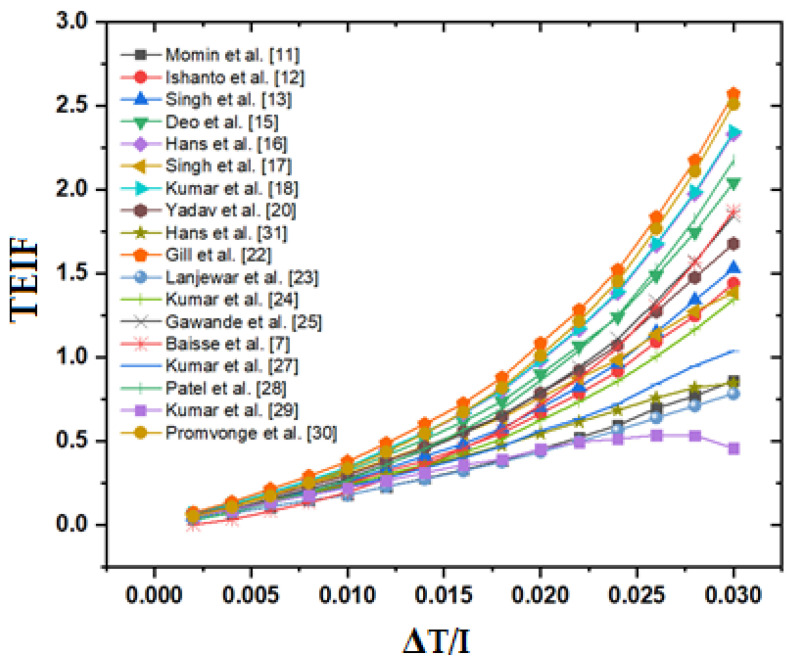
TEIF of the roughened duct with respect to ΔT/I.

The plot between the thermal efficiency improvement factor (TEIF) and ΔT/I is shown in Figure 7. The TEIF shows augmentation in the thermal performance due to roughness as a comparison to a smooth surface [42]. The TEIF is expressed as:

(34) $$TEIF=\frac{{\left(\eta_{th}\right)}_{r}-{\left(\eta_{th}\right)}_{s}}{{\left(\eta_{th}\right)}_{s}}$$

The twisted rib and W-shaped rib show a lower value of TEIF while the staggered broken arc hybrid-rib roughness shows higher values of TEIF consistently among all other distinct roughness geometries for the ascending values of ΔT/I. On the basis of the TEIF value of distinct roughness, it can be stated that the staggered broken arc hybrid-rib roughness performs better as a comparison to others with a simultaneous increase in consumption of pumping power.

**Figure 8:**
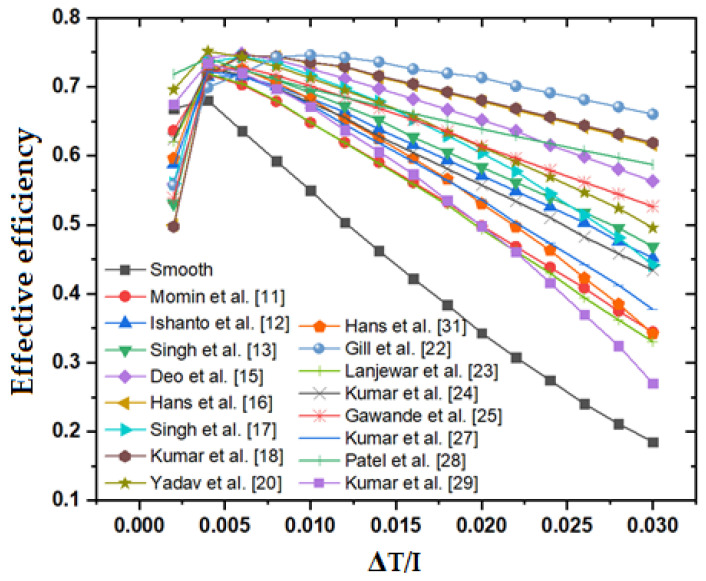
Effective efficiency of the roughened duct with respect to ΔT/I.

The effective efficiencies of distinct roughness are plotted as a function of the rising value of ΔT/I in Figure 8 to indicate the real improvement in performance with a simultaneous increase in pumping power consumption. 

Table 3 contains information on effective efficiency. Among all different roughness, the effective efficiency initially achieves a maximum value and then decreases further increases in ΔT/I. All individual roughness’s in the ΔT/I range of 0.0015 to 0.0100 achieve the best effective efficiency value. In comparison to other roughness, the staggered broken arc hybrid-rib roughness performs better.

The thermal and effective efficiency of the rough surfaces of various ribs configurations has been compared with the thermal and effective efficiency of the smooth surface for the same ranges of operating parameters. It was found that hybrid-rib of broken arc and staggered rib shows the highest thermal as well as effective efficiency. Similarly, TEIF shows higher values in the case of e/D_h_ = 0.043 and α = 60°. The thermal and effective efficiency also shows higher values at these parameters. It is found that most of the rib roughness geometries have optimum performance at p/e = 10, W/w = 6, e/D_h_ = 0.043 and α = 60°. The highest value of effective efficiency is achieved by all distinct roughnesses in the ΔT/I range from 0.0015 to 0.0100. Moreover, the unique combination of parameters p/e = 10, e/D_h_ = 0.043 and α = 60° are observed for the best performance at a ΔT/I higher than 0.00789 K·m^2^/W.

## 5. Conclusions

This study deals with presenting and discussing methods proposed to improve the performance of the basic configuration of the solar air heaters (SAH). A general approach based on developing a thermo-hydraulic mathematical model for a modified SAH and integrating various heat transfer and friction factor correlations from different previous studies has been proposed and employed. A numerical study has been carried out to predict the thermo-hydraulic performance of roughened absorber surface of SAH on the basis of thermal and effective efficiency. The thermal and effective efficiency of the rough surfaces also has been compared with the thermal and effective efficiency of the smooth surface in the same experimental conditions. The main findings are highlighted as follows:

(a) The shape, size, and arrangement of artificial roughness on the duct surface are the most important factors for the performance optimization of SAH.
(b) The broken-arc shaped ribs, combined with staggered-arc rib pieces have better performance over the broken arc shape and arc shape rib roughness.
(c) The thermal and effective efficiency strongly depends on the roughness parameter, Re, and ΔT/I.
(d) For most of the rib roughness geometries, optimum performance has been achieved at the following parameters; p/e = 10, W/w = 6, e/D_h_ = 0.043 and α = 60°.
(e) The highest value of effective efficiency is achieved by all distinct roughnesses in the ΔT/I range of 0.0015 to 0.0100.
(f) The twisted rib shows a lower value of effective efficiency at a higher value of ΔT/I.
(g) The unique combination of parameters p/e = 10, e/d_h_ = 0.043 and α = 60° are observed for the best performance at ΔT/I > 0.00789 K·m^2^/W.
(h) The staggered broken arc hybrid-rib roughness consistently shows a higher thermal efficiency among all other distinct roughness geometries for the ascending values of ΔT/I.
(i) The staggered broken arc hybrid-rib roughness shows higher effective efficiency over the other roughnesses. This roughness shows a value of effective efficiency equal to 74.63% at ΔT/I equal to 0.01.
(j) The staggered broken arc hybrid-rib roughness consistently shows higher values of TEIF among all other distinct roughness geometries for the ascending values of ΔT/I.

## Figures and Tables

**Table 1:** Value/range of system and operating parameter.

Parameter	Value/Range
**System Parameters** *Fixed*	
Collector duct height (H), m	0.025
Collector length (L), m	1.0
Collector width (W), m	0.3
Emissivity of absorber plate (ε_p_)	0.9
Emissivity of transparent glass cover (ε_g_)	0.88
Gap between collector and glass cover (L_g_)	0.025
Number of glass cover (N)	1
Thickness of back insulation (t_i_), m	0.05
Thermal conductivity of insulation (k_i_), W/m·K	0.037
Thickness of collector edge (t_e_), m	0.1
Thickness of glass cover (t_g_), m	0.002
Tilt angle (β_ti)_	30°
Transmittance-absorptance product	0.8
*Variable*	
Relative rib height (e/D_h_)	0.020–0.044
Relative rib pitch (p/e)	6–12
**Operating parameters**	
*Fixed*	
Ambient temperature (T_a_), K	285
Wind velocity (V_w_), m/s	1.0
*Variable*	
Insolation (I), W/m^2^	600–1000
Temperature rise parameter (ΔT/I), K·m^2^/W	0.002–0.030

**Table 2:** Correlations developed by researchers for different roughened surfaces.

Investigators	Roughness	Correlations
Baissi et al. [7]	Delta shape tubulators	Nu=0.5884Re0.4793(Pl/e)0.5943(Pt/b)−0.3201×exp[−0.5426{ln(Pl/e)}2] f=0.338Re−0.0996exp[−1.2539{ln(Pl/e)}2]×(Pl/e)2.1042×(Pt/b)−0.56exp[−0.2375{ln(Pt/b)}2]
Ebrahim-Momin et al. [11]	V-shape continuous ribs	Nu=0.067Re0.888(α/60)−0.077(e/Dh)0.424exp[−0.728{ln(α/60)}2] f=6.266Re−0.425(α/60)−0.093(e/Dh)0.565exp[−0.719{ln(α/60)}2]
Istanto et al. [12]	V-shape rib	Nu=0.016Re0.891(α/90)−1.123exp[−1.107{ln(α/90)}2] f=31.589Re−0.759(α/90)−1.385exp[−1.318{ln(α/90)}2]
Singh et al. [13]	V-shape with gap	Nu=2.36×10−3(p/e)3.50Re0.90(e/Dh)0.47(d/w)−0.043(α/60)−0.023(g/e)−0.014exp[−0.84{ln(p/e)}2]×exp[−0.05{ln(d/w)}2]×exp[−0.72{ln(α/60)}2]×exp[−0.15{ln(g/e)}2] f=4.13×10−2Re−0.126(e/Dh)0.7(p/e)2.74(d/w)−0.058(α/60)−0.034(g/e)0.31exp[−0.685{ln(p/e)}2]×exp[−0.058{ln(d/w)}2]×exp[−0.93{ln(α/60)}2]×exp[−0.21{ln(g/e)}2]
Deo et al. [15]	Multi-gap-V-down rib	Nu=0.02253Re0.98(e/Dh)0.18(p/e)−0.06(α/60)0.04 f=0.3715Re−0.15(e/Dh)0.65(p/e)0.21(α/60)0.57
Hans et al. [16]	Continuous multi-V-ribs	Nu=3.35×10−5Re0.92(e/Dh)0.77(p/e)8.54(α/90)−0.49(W/w)0.043×exp[−0.61{ln(α/90)}2]×exp[−2.0407{ln(p/e)}2]×exp[−0.1177{ln(W/w)}2] f=4.47×10−4Re−0.3188(p/e)8.9(e/Dh)0.73(α/90)−0.39(W/w)0.22×exp[−0.52{ln(α/90)}2]×exp[−2.133{ln(p/e)}2]×exp[−0.1177{ln(W/w)}2]
Singh et al. [17]	Multi V-ribs with uniform gap	Nu=0.0187Re1.176×(p/e)−0.6586×(e/Dh)0.4927×(e/w)0.033×(W/w)0.0659×(g/e)0.1753×(x/w)0.1147×exp[−0.011{ln(W/w)}2]×exp[0.1837{ln(p/e)}2]×exp[0.0775{ln(x/w}2]×exp[0.0869{ln(e/w)}2]×exp[0.2413{ln(g/e)}2] f=1.3601Re−0.434×(p/e)−0.7032×(e/Dh)0.0863×(e/w)0.0229×(W/w)0.0858×(g/e)0.1436×(x/w)−1.3003×exp[0.0098{ln(W/w)}2]×exp[0.1925{ln(p/e)}2]×exp[−0.4762{ln(x/w}2]×exp[−0.0556{ln(e/w)}2]×exp[0.0455{ln(g/e)}2]
Kumar et al. [18]	Multi V-ribs with gap	Nu=8.532×10−3Re0.932(e/Dh)0.175(p/e)1.196(α/60)−0.0239(g/e)−0.0708(W/w)0.506(Gd/Lv)−0.0348exp[−0.2805{ln(p/e)}2]×exp[−0.0753{ln(W/w)}2]×exp[−0.1153{ln(α/60)}2]×exp[−0.0653{ln(Gd/Lv)}2]×exp[−0.223{ln(g/e)}2] f=3.1934Re−0.3151(e/Dh)0.268(α/60)0.1553(p/e)−0.7941(g/e)−0.1769(W/w)0.1132(Gd/Lv)0.0610exp[0.1486{ln(p/e)}2]×exp[0.0974{ln(W/w)}2]×exp[−0.1527{ln(α/60)}2]×exp[−0.1065{ln(Gd/Lv)}2]×exp[−0.6349{ln(g/e)}2]
Yadav et al. [20]	Dimple ribs in arc arrangement	Nu=0.154Re1.017(e/Dh)0.521(p/e)−0.38(α/60)−0.213×exp[−2.023{ln(α/60)}2] f=7.207Re−0.56(e/Dh)0.176(p/e)−0.18(α/60)−0.038×exp[−1.412{ln(α/60)}2]
Gill et al. [22]	Hybrid-rib	Nu=3.596×10−3Re1.068(g/e)−0.018(r/e)−0.02(W1/w)−0.073(p/e)1.403(α/90)−0.408(e/Dh)0.56×exp[−0.151{ln(g/e)}2] f=7.981×10−2Re−0.157(g/e)−0.021(r/e)0.012(W1/w)−0.104(p/e)1.739(α/90)−0.638(e/Dh)0.783
Lanjewar et al. [23]	Continuous W-ribs	Nu=0.0613Re0.9079(α/60)−0.1331(e/Dh)0.4487×exp[−0.5307{ln(α/60)}2] f=0.06182Re−0.2554(α/60)0.0817(e/Dh)0.4682×exp[−0.28{ln(α/60)}2]
Kumar et al. [24]	Discrete W-ribs	Nu=0.105Re0.873(α/60)−0.081(e/Dh)0.453×exp[−0.59{ln(α/60)}2] f=5.86Re−0.40(α/60)0.081(e/Dh)0.59×exp[−0.579{ln(α/60)}2]
Gawande et al. [25]	S-shape rib	Nu=0.032(p/e)0.3479Re0.8332exp{−0.1004ln(p/e)2} f=0.280(p/e)0.0815Re−0.2617exp{−0.0319ln(p/e)2}
Kumar and Layek [27]	Winglet turbulators	Nu=3.64×10−5Re0.95(α/75)−0.91(p/e)3.73(W/w)2.37×exp[−0.90{ln(p/e)}2]×exp[−1.22{ln(α/75)}2]×exp[−0.81{ln(W/w)}2] f=0.13Re−0.37(W/w)2.41(α/75)−0.45(p/e)−0.12×exp[−0.77{ln(W/w)}2]
Patel et al. [28]	NACA 0040 profile rib	Nu=0.009016(e/Dh)−3.1354×Re0.526exp[−0.5834{ln(e/Dh)}2] f=0.32449Re1.3728×(e/Dh)5.6236×exp[0.943{ln(e/Dh)}2]×exp[−0.0875{ln(Re)}2]
Kumar and Layek [29]	Twisted tape	Nu=3×10−10Re1.043(y/e)−0.17(p/e)15.75(α/60)−0.84×exp[−3.75{ln(p/e)}2]×exp[−0.85{ln(α/90)}2] f=6.82Re−0.58(y/e)0.31(α/60)0.23(p/e)−0.42
Promvonge et al. [30]	Combination of V-shape rib and delta groove	Nu=1.48Re0.537Pr0.4(p/H)−0.269(e/H)0.126 f=6.8Re0.127Pr0.4(p/H)−0.521(e/H)1.096
Hans et al. [41]	Broken arc rib	Nu=1.014×10−3(Re)1.036(d/w)−0.078(e/Dh)0.412(p/e)2.522(α/90)−0.293(g/e)−0.016×exp[−0.114{ln(α/90)}]2×exp[−0.567{ln(p/e)}2]exp[−0.133{ln(g/e)}2]×exp[−0.077{ln(d/w)}2] f=8.1921×10−2(Re)−0.147(e/Dh)0.528(α/90)−0.292(p/e)1.191(d/w)−0.067(g/e)−0.006×exp[−0.110{ln(α/90)}]2×exp[−0.255{ln(p/e)}2]exp[−0.158{ln(g/e)}2]×exp[−0.063{ln(d/w)}2]

**Table 3:** Effective efficiency data at distinct value of ΔT/I.

ΔT/I	0.002	0.004	0.006	0.008	0.01	0.012	0.014	0.016	0.018	0.02	0.022	0.024	0.026	0.028	0.03
Smooth	0.66868	0.68033	0.63622	0.59254	0.54978	0.50358	0.46201	0.42204	0.38383	0.34295	0.30766	0.27455	0.24051	0.21156	0.18508
Momin et al. [11]	0.63675	0.71773	0.70303	0.67906	0.64814	0.61951	0.59057	0.56152	0.53248	0.49855	0.46833	0.43823	0.40836	0.3747	0.34462
Ishanto et al. [12]	0.58813	0.71467	0.71596	0.70154	0.68322	0.66356	0.6385	0.6164	0.59402	0.57146	0.54877	0.526	0.5032	0.4757	0.45193
Singh et al. [13]	0.53042	0.72252	0.72528	0.71013	0.69165	0.67222	0.65247	0.62694	0.60533	0.58355	0.56163	0.53961	0.51751	0.49537	0.46835
Deo et al [15].	0.5585	0.74153	0.7483	0.73928	0.72627	0.71224	0.69762	0.68263	0.66738	0.6519	0.63624	0.61547	0.59828	0.5808	0.56304
Hans et al. [16]	0.49956	0.72128	0.74573	0.74358	0.73512	0.7289	0.71403	0.70247	0.69062	0.67858	0.66638	0.65404	0.64157	0.62899	0.6163
Singh et al. [17]	0.55994	0.73565	0.74361	0.73529	0.71674	0.69842	0.67871	0.65325	0.62932	0.60407	0.57755	0.5452	0.51417	0.48138	0.4416
Kumar et al. [18]	0.49731	0.71725	0.74524	0.74419	0.73548	0.72947	0.71574	0.70436	0.69268	0.68076	0.66868	0.65644	0.64407	0.63156	0.61893
Yadav et al. [20]	0.69573	0.7516	0.74318	0.72971	0.71291	0.69518	0.6769	0.65822	0.63397	0.61293	0.59138	0.56935	0.54686	0.52396	0.49576
Hans et al. [31]	0.59659	0.73088	0.72602	0.70595	0.68272	0.65305	0.62513	0.59616	0.5663	0.53068	0.49732	0.46297	0.42293	0.38508	0.34181
Gill et al. [22]	0.55736	0.6994	0.71942	0.74323	0.74632	0.74269	0.73627	0.72566	0.71974	0.71344	0.70123	0.6914	0.68131	0.67097	0.6604
Lanjew et al. [23]	0.62051	0.71927	0.70464	0.6801	0.64839	0.61892	0.58905	0.55898	0.52884	0.49308	0.46141	0.42979	0.39439	0.36214	0.33027
Kumar et al. [24]	0.62424	0.72688	0.71691	0.69814	0.67719	0.65569	0.62819	0.60472	0.58116	0.55757	0.53399	0.51045	0.48255	0.45822	0.43397
Gawan et al. [25]	0.53892	0.72154	0.72854	0.71667	0.7014	0.68519	0.6687	0.65215	0.63562	0.61416	0.59669	0.5792	0.56171	0.54422	0.52674
Kumar et al. [27]	0.58445	0.72223	0.7163	0.69693	0.67431	0.64529	0.61876	0.59165	0.56411	0.53626	0.50293	0.47308	0.44295	0.41266	0.37736
Patel et al. [28]	0.71828	0.74133	0.72515	0.7107	0.69734	0.68476	0.67275	0.66117	0.64996	0.63904	0.62835	0.61787	0.60756	0.5974	0.58735
Kumar et al. [29]	0.67466	0.7342	0.72038	0.69722	0.67142	0.63736	0.60608	0.57363	0.53499	0.49831	0.46042	0.41577	0.36973	0.32461	0.27022

## Data Availability

Not applicable.

## Nomenclature

**Symbol**	**Title**	**Unit**
b	Roughness width	m
c	Characteristic separation length	m
d′	Diameter of dimples	m
d/x	Relative gap position	-
d_R_	Relative punched hole size	-
D	Pipe inside diameter (to base of ribs)	m
D_e_	Equivalent diameter of annulus (d_1_-d_2_)	m
D_h_	Hydraulic diameter of duct	m
e	Roughness height	m
e^+^	Roughness Reynolds number	-
e/D_h_	Relative roughness height	-
e/H	Blockage ratio	-
*f_s_*	Friction factor of smooth surface	-
*f*	Friction factor of roughened surface	-
F_R_	Heat removal factor	-
g	Heat transfer roughness function	-
hr	Radiative heat transfer coeff.	W/m^2^·K
hc	Convective heat transfer coeff.	W/m^2^·K
hw	Wind convective heat transfer coeff.	W/m^2^·K
I	Insolation	W/m^2^
L	Length of test section	m
Nu	Nusselt number	-
N_g_	Number of gaps on half arc	-
p/g	Relative pitch to gap ratio	-
p/H	Rib pitch to channel height ratio	-
p′/p	Relative staggered rib pitch	-
P_t_/b	Relative longitudinal length of obstacles	-
P_t_/e	Relative transversal length of obstacles	-
P_r_	Prandtl number	-
P_R_	Relative winglet pitch	-
Q_u_	Heat gain	W
Qe^+^	Heat transfer function	-
r/e	Relative staggered rib size	-
R	Momentum transfer roughness function	-
Ra	Rayleigh number	-
Re	Reynolds number	-
S	Short way length between dimples	m
s′/s	Relative gap position	-
St	Stanton number	-
T_a_	Ambient temperature	K
T_f_	Mean air temperature	K
T_i_	Air inlet temperature	K
T_p_	Plate temperature	K
T_w_	Wall temperature	K
ΔP	Pressure drops	N/m^2^
U_O_	Overall heat loss coefficient	W/m^2^·K
V	Velocity of air in SAH duct	m/s
w/e	Staggered rib length to rib height	-
W/e	Width to height ratio	-
W/H	Width to duct height ratio	-
W_1_/w	Relative gap position	-
W/w	Relative roughness width	-
t_i_	Thickness of insulation	mm
t_g_	Thickness of glass cover	mm
t_g_	Height of collector edge	mm
U_b_	Back heat loss coefficient	W/m^2^·K
U_e_	Edge heat loss coefficient	W/m^2^·K
U_t_	Top heat loss coefficient	W/m^2^·K
V	Air velocity	m/s
V_w_	Wind Speed	m/s
α	Angle of attack	degree
α′	Arc angle	degree
α/90	Relative arc angle	-
*φ*	Chamfering angle of rib	degree
Β	Slope	degree
β′	Thermal expansion coefficient of air	1/K
ε_g_	Glass cover emissivity	
ε_p_	Absorber plate emissivity	
υ	Kinematic viscosity,	m^2^/s
τ	Transmissivity	
σ	Steafan-Boltzmann constant,	W/m^2^·K^4^
η	Thermal efficiency	-
